# Hepatoprotective and antioxidant effects of single clove garlic against CCl_4_-induced hepatic damage in rabbits

**DOI:** 10.1186/s12906-017-1916-8

**Published:** 2017-08-17

**Authors:** Khalid Mohammed Naji, Elham Shukri Al-Shaibani, Fatima A. Alhadi, Safa’a Abdulrzaq Al-Soudi, Myrene R. D’souza

**Affiliations:** 1 Biochemistry section, Department of Chemistry, Faculty of science, Sana’a University, P.O. Box 13499, Sana’a, Yemen; 2 Zoology section, Department of Biology, Faculty of Science, Sana’a University, Sana’a, Yemen; 3 Botany section, Department of Biology, Faculty of Science, Sana’a University, Sana’a, Yemen; 4 Department of Biochemistry, Mount Carmel College, No.58, Palace Road, Vasanth Nagar, Bengaluru, Karnataka 560052 India

**Keywords:** *Allium sativum*, Antioxidant, Biochemical parameters, Carbon tetrachloride, Hepatotoxicity, Single clove garlic, Oxidative toxicity

## Abstract

**Background:**

The increase in demand and consumption of single clove garlic or ‘Solo garlic’ (*Allium sativum*) has resulted in an increase in research on its therapeutic properties. The present study aims to evaluate the antioxidant activities, oxidant-scavenging efficiency and preventive effects of SCG (single clove garlic) and MCG (multi clove garlic) on CCl_4_-induced acute hepatotoxicity in male rabbits.

**Methods:**

For this purpose, rabbits were orally administered with 3 ml of CCl_4_ /kg of body weight, followed by 0.8 g of MCG or SCG/kg twice a week for three successive weeks. Oxidative hepatotoxicity was then assessed.

**Results:**

SCG extracts exhibited higher antioxidant capacity than the MCG extract. Scavenging ability of SCG showed significant (*p* < 0.05) elevation against 2,2-diphenyl-1-picrylhydrazyl (DPPH) and superoxide radicals in comparison to MCG. In addition, total phenolic content of SCG was significantly elevated (*p* < 0.001), thereby suggesting that the composition of garlic storage constituents varies with the number of cloves present. CCl_4_-induced hepatotoxicity demonstrated histological changes including severe damage in the structure of liver tissues which correlated well to oxidative stress levels. Simultaneously, administration of SCG resulted in a significant reduction of serum alkaline phosphatase (ALP), aspartate aminotransferase (AST), alanine aminotransferase (ALT), and total bilirubin (TB) levels in addition to improvement in some histological parameters. Low levels of lipid peroxidation (malondialdehyde, MDA) (*p* < 0.001), along with a huge reduction in peroxidase (POx) (*p* < 0.001) revealed protection against oxidative toxicity in the liver homogenate. Higher levels of catalase (CAT) (*p* < 0.001) and superoxide dismutase (SOD) (*p* < 0.05) when compared to the MCG test (TM) group indicates that removal of H_2_O_2_ is based on CAT activity in SCG test (TS) group rather than the POx activity demonstrated in the former group.

**Conclusion:**

The present study indicates that SCG possesses more protective ability than MCG against CCl_4_-induced liver injury and might be an effective alternative medicine against acute oxidative liver toxicity.

**Electronic supplementary material:**

The online version of this article (doi:10.1186/s12906-017-1916-8) contains supplementary material, which is available to authorized users.

## Background

Toxicity is the ability of a substance to cause harmful effects on a single cell, a group of cells, an organ system, or the entire body [1]. Many chemicals found in the environment are toxic and necessitate accurate identification of their potential hazards to both human and animal health. Among various chemicals that injure the organs, carbon tetrachloride (CCl_4_) is found to be the most toxic [2]. CCl_4_ is an extensively used chemical solvent in various industrial processes. It is a well-established hepatotoxic substance and is the best-characterized animal model of xenobiotic-induced free radical-mediated hepatotoxicity [3]. The metabolism of CCl_4_ in animal cells induces ROS formation through trichloromethyl radical via the cytochrome P450 enzyme system [4]. The radicals thus formed increases lipid metabolism with a consequent decrease in its transport out of the hepatocyte causing steatosis, or fatty liver. Reactive aldehydes formed by breakdown of radicals increase membrane permeability and finally cause cell death. Dominance of such pro-oxidants over antioxidants causes oxidative stress leading to chemical modification and/or damage of proteins, carbohydrates, nucleotides and lipids [5]. Liver sections of rabbits treated with CCl_4_ show total loss of hepatic architecture with massive fatty changes, congestion of sinusoids, intense necrosis, and infiltration of the lymphocytes around the central vein [6]. In addition, hepatocyte necrosis, hemorrhage, vacuolar change and hydropic degeneration were apparent in mice after CCl_4_ administration [7].

Liver, the key homeostatic organ involved in most biochemical processes also detoxifies harmful drugs and chemicals. With the rise in cases of jaundice and hepatitis, development of hepatoprotective drugs from natural sources has become necessary. Hence, there is a need to review traditional systems of medicine for remedies to common hepatic disorders. Garlic (*Allium sativum*) is one such herb commonly consumed in foodstuffs and used in nutraceuticals. It is comprised of bulbs called cloves formed by two types of bulbing processes – in the first type, there is formation of lateral buds in the axils of some of the youngest leaves. This normally occurs with inflorescence formation, often producing multi clove bulbs. In the second type, a transition of lateral buds into storage leaves occurs, and the sprout enclosed within the storage leaf gradually becomes dormant as the storage leaf grows. This process often produces a single clove bulb [8]. The latter form of bulb is usually separated during harvesting and packed as a single clove garlic (SCG), while the former is sold as multi glove garlic (MCG).

Garlic is known for its antidiabetic, anticancer, antioxidant, immune modulation activities [9–11]. It has been shown to inhibit LPO and dose-dependent induction of endogenous antioxidants in rat kidney and liver [12]. The higher concentration of organosulfur compounds in garlic, in comparison to other Allium species is responsible for both garlic’s pungent odor and flavor as well as many of its medicinal effects [13]. Alliinase acts on alliin (S-allylcysteine sulfoxide) to produce an antioxidant compound, allicin, known to scavenge hydroxyl radicals and inhibit LPO [14]. In addition, the presence of thiosulfinates and other secondary metabolites, including steroids, terpenoids, flavonoids and other phenols, may be responsible for reported therapeutic effects of garlic [15].

In addition, garlic components were able to increase the activities of antioxidant enzymes such as catalase, superoxide dismutase, glutathione peroxidase and glutathione-s-transferase [11, 16]. Thus, garlic is used as an alternative remedy for the treatment of various infections; to lower blood pressure, cholesterol and sugar, prevent blood clotting, boost the immune system and maintain overall health [17, 18].

Garlic has been studied in different forms of extracts: aqueous, ethanol, dried powders. Garlic extracts have been shown to attenuate hyperhomocysteinemia caused by folic acid deficiency in rats [19] or gentamicin-induced renal damage and oxidative stress in rats [20]. In addition, garlic powder supplemented diet has demonstrated protective effects in cardiac ischemia and reperfusion [21], adriamycin-induced toxicity [22], azoxymethane-induced damage [23], and hypercholesterolemic and high fat [24] diet-induced oxidative damage. Moreover, garlic has chemopreventive potential against cyclophosphamide induced chromosomal mutations in Swiss albino mice [25].

In the present communication, we extend observations on CCl_4_-induced liver damage in rabbits, an animal model for hepatotoxicity and report experiments on hepatocytes exposed to CC1_4_, with the ensuing toxicity modified by SCG possibly targeting different steps of the CC1_4_ attack. Several typical parameters of CC1_4_ toxicity were monitored in the absence and presence of garlic supplementation. The results contribute to a better understanding of the mechanism of CC1_4_-induced steatosis and hepatotoxicity.

## Methods

### General

FC reagent, 2,2-Diphenyl-1-picrylhydrazyl (DPPH), nitro blue tetrazolium (NBT), reduced glutathione (GSH), thiobarbituric acid (TBA), ascorbic acid, 5,5′-dithio-bis-[2-nitrobenzoic acid] (DTNB), methanol and ethanol were purchased from Sigma-Aldrich Chemical Company (St. Louis, MO, USA). Potassium dihydrogen phosphate (KH_2_PO_4_), ethylenediaminetetra acetic acid (EDTA), 1-chloro-2,4-dinitrobenzene (CDNB), sulfuric acid (H_2_SO_4_), and glacial acetic acid were purchased from British Drug Houses (BDH), UK. Hydrogen peroxide (H_2_O_2_), gallic acid and were obtained from Merck Chemicals, USA.

### Plant material

Bulbs of SCG and MCG were collected during the month of August/September from a local market at Bani-Matar district near Sana’a city, Yemen (Geographic coordinate: latitude 15°15′10.43″N and longitude 44° 1′23.72″E). A voucher specimen (No. 00356) of SCG and MCG have been deposited in the herbarium at the department of Biology, Sana’a University which is available for the public (Additional file 1: Figure S1 and Additional file 2: Figure S2).

Garlic samples were cut into small pieces to ease the drying process. The dried garlic was pulverized using a mortar and pestle followed by extraction with 70% ethanol in Soxhlet apparatus for 12 h at 60 °C. The solvent was removed by rotary evaporation at 50 °C and then subjected to freeze drying to remove moisture. The lyophilized powder was stored in the dark at 4 °C. For animal administration, the powder was suspended in distilled water and was orally administered via gastric tube.

### Measurement of total phenolic content

Total phenolic content (TPC) of the extracts of MCG and SCG were determined using the method of Singleton [26] with some modification. The reaction mixture contained 0.5 ml of test sample (0.1 g/ml) and 1.5 ml of 10% diluted Folin-Ciocalteau reagent. It was incubated for 5 min at 25 °C. This was followed by addition of 2.0 ml of 7.5% of sodium carbonate and then incubated in the dark for 90 min with intermittent shaking. Absorbance of samples were measured at 725 nm using spectrophotometer. The total phenolic contents were calculated based on gallic acid standard curve (0 –50 μg/mL) and were expressed as gallic acid equivalents GAE/g of the extract.

### In vitro measurement of plant scavenging activity

#### 2,2-diphenyl-1-picrylhydrazyl (DPPH) radicle

The scavenging ability of the natural antioxidants of the SCG and MCG extract towards the stable free radical DPPH was measured [27] with slight modification. 10, 20 μl of the extract (0.1 g/ml) were added to a methanolic solution of 0.3 mM DPPH up to a final volume of 0.5 ml. The mixture was incubated at room temperature for 30 min and the discoloration of the purple color spectroscopically measured at 518 nm. Methanol and DPPH in methanol (without extracts) served as blank and positive control respectively. The radical scavenging activity was calculated using the following equation:

$$ DPPH radical scavenged\ \left(\%\right)={OD}_{Control}-{OD}_{Sample}/{OD}_{Control}\times 100 $$

### Superoxide radical

Measurement of the superoxide anion scavenging activity of the sample was performed and measured spectrophotometrically at 560 nm [28]. Superoxide scavenging activity was calculated using the following equation:

$$ Superoxide scavenging activity\ \left(\%\right)=\left({A}_C-{A}_S/{A}_C\right)\times 100 $$

where: *A*_*C*_ is the absorbance of the blank and *A*_*S*_: is the absorbance of test sample. Ascorbic acid was used as a positive control.

### Hydrogen peroxide scavenging activity

The ability of the extracts to scavenge hydrogen peroxide was assessed by the method of Ruch et al., [29]. 10 and 20 μl of the extracts (0.1 g/ml) were added to 0.6 ml of 40 mM H_2_O_2_ solution prepared in 0.1 M phosphate buffer (pH 7.4). The total volume was made up to 3 ml with buffer, incubated for 10 min and the absorbance read at 230 nm using UV spectrophotometer against a buffer blank. The control was similar to the reaction mixture but without H_2_O _2_. The scavenging activity was calculated using the following equation:

$$ {H}_2{O}_2\kern0.5em Scavenging activity\ \left(\%\right)={A}_{reaction mixture}/{A}_{control}\times 100 $$

### Experimental animals

Twenty-four healthy domestic male rabbits (*Oryctolagus cuniculus domesticus*) (750 – 900 g) were purchased from the local market, Sana’a. The rabbits were housed in the animal house of the Biology Department, Faculty of Science, Sana’a University, Sana’a. They were acclimatized for 2 weeks prior to experimentation. This was done to enable adaptation to the surroundings and enforce a daily routine with 12 to 13 h of light to maintain the colony’s circadian biorhythms. The diet consists of fresh hay, water, and fresh vegetables. The rabbits were routinely observed for food consumption and fecal characteristics. Water was changed every day and available around the clock.

### Experimental design

The rabbits were randomly divided into four groups, each comprising of six rabbits. Each rabbit was placed individually in a separate mesh cage (0.90 × 0.60 × 0.40 m) with standard laboratory diet and water ad libitum*.* The cages were provided with collection trays below the ground mesh. The first group Control (C), received olive oil 3 ml/kg twice a week orally. The second (T), third (TS) and fourth (TM) groups orally received 3 ml/kg of CCl_4_ in olive oil (1: 1) as a 1/4 LD_50_, [30] twice a week for three successive weeks. After 30 min of the ingestion, the third group (TS) and fourth group (TM) was treated with 0.8 g lyophilized SCG and MCG extract/kg of body weight respectively. The animals were maintained at almost constant environmental conditions during the experiment period.

All experiments in this study were performed three times with 24 months during 2013-2015.

### Collection of blood and tissue samples

Twenty-four hours after the completion of the experimental period, all rabbits were subjected to overnight fasting, anesthetized by slow administration of Ketamine/xylazine (60/8 mg/kg) through the lateral auricular vein. After the anesthesia, at 09:15 AM rabbits were sacrificed by cutting the neck using a sharp blade and dissected in the animal anatomy laboratory. Blood was collected from each rabbit in a collection tube with activated gel and sent for liver function analysis. Immediately after blood collection, the liver of each rabbit was removed and fixed for histological study. Small parts of liver were immediately cut out, washed with ice-cold normal saline, and stored at −20 °C for biochemical analysis.

### Measurement of serum ALT, AST, ALP, TB and TSP

Serum ALT, AST, ALP, TB, and TSP were determined by kinetic UV assay using kits supplied by Roche Diagnosis attached with Roche/Hitachi Analyzer machine at Al-Aulaqi Specialized Medical Laboratory, Sana’a.

### Histopathological analysis

For histological analysis, liver specimens were washed in normal saline and fixed with 10% formalin. Fixed tissues were embedded in paraffin wax, sectioned in rotary microtome (5 μm thick) and then stained with haematoxylin and eosin dyes [31]. Tissue samples were coded and evaluated for any histological changes using a light microscope (Leica Galen III). and photographed by a digital camera.

Quantitative analysis of the histopathological changes in liver was calculated from an average obtained from observation of six rabbits per group using an ocular micrometer calibrated with a stage micrometer. Evaluating the frequency of the histopathological changes was obtained based on average from observation of 12 microscope fields with an area 625μm^2^ at 40X or 400X.

### Measurement of lipid peroxidation

Malondialdehyde (MDA) levels were estimated by its reaction with thiobarbituric acid to form a complex called thiobarbituric acid reactive substance (TBARS) that absorbs at 535 nm [32]. The values were expressed as nmol/mg tissue.

### Measurement of glutathione levels

Reduced glutathione reacts with 5,5′-dithiobis-2-(nitrobenzoic acid) (DTNB) to produce a yellow colored product that absorbs at 412 nm [33]. A calibration curve was performed with standard GSH (2-10 nmole) and concentration was expressed as nmole GSH/mg tissue.

### Measurement of superoxide dismutase

SOD (EC: 1.15.1.1) activity was determined with some modification [34]. One unit of SOD is defined as the amount of enzyme causing 50% inhibition in the nitro blue tetrazolium (NBT) reduction rate. SOD activity was also expressed as U/ mg protein.

### Catalase

CAT (EC: 1.11.1.6) activity was determined according to the method of Aebi et al., [35]. The principle of this method is based on the determination of decomposition rate H_2_O_2_ at 240 nm. The results were expressed as U/mg protein.

### Peroxidase

POx (EC: 1.11.1.7) activity was estimated using guaiacol as substrate for the assay [36]. The increase in the absorption as a result of the formation of the oxidized product tetraguaiacol was measured at 470 nm using the extinction coefficient of 26.6 mM^−1^ cm^−1^.

### Measurement of glutathione-s-transferase

GST (EC: 2.5.1.18) activity was assayed by measuring conjugated GSH and CDNB, the extent of conjugation causing a proportionate change in the absorbance at 340 nm [37]. The reaction mixture contained phosphate buffer (50 mM), CDNB (200 mM), GSH (200 mM), and 10 μL samples. The reaction was carried out at 37 °C and the increase in absorbance of the product was monitored using UV-VIS Spectrophotometer (Specord200, Analytikjena, Germany). A blank was run in absence of the sample. One unit of GST activity was expressed as μmoles of CDNB conjugated/min.

### Total protein (TP)

Amount of total protein in tissue was determined by Lowry et al., [38].

### Statistical analysis

All presented data were expressed as a mean ± SD of three separated repeats. The statistical significances among groups was analysed using one-way analysis of variance (ANOVA) followed by Tukey Multiple Comparisons with the help of Prism 6 software (GraphPad, San Diego, CA, USA). A value of *p* < 0.05 was considered significant.

## Results

### In vitro antioxidant activity of garlic extract

A comparison of the antioxidant capacities of lyophilized powders of ethanolic extracts showed greater scavenging ability by SCG against DPPH and superoxide radical (Table 1). Both SCG and MCG extracts exhibited a high ability to scavenge H_2_O_2_, which was equal to or more than the scavenging ability of ascorbic acid, commonly used as standard antioxidant (Table 1).

**Table 1:** Radical scavenging ability of SCG extracts against different oxidants^a^

	% of radical scavenging activity		
	DPPH	H_2_O_2_	${\mathrm{O}}_2^{\bullet -}$
AsA (Control)	94.13 ± 0.2192	95.88 ± 1.075	57.438 ± 2.06
SCG	50.70 ± 0.567 ^b c^	96.35 ± 0.622	23.67 ± 14.7
MCG	36.93 ± 1.414	95.68 ± 1.584	20.54 ± 9.7

Each value presented is mean ± SD of three repetitions

^a^Concentration of the extracts used were 0.1 g/ml

^b,c^ *p* < 0.05, vs MCG

*SCG* single clove garlic, *MCG* multi clove garlic, *AsA* ascorbic acid

Total phenolic content (TPC) of plants extracts serve as a positive indicator for natural antioxidant sources. TPC levels are directly proportional to the antioxidant activity of the sample. The elevation of TPC in SCG was significantly higher (*p* < 0.001) at 3.5 folds when compared to the levels in MCG (Table 2).

**Table 2:** Comparison of the total phenol content (TPC) of MSG and SCG extracts^a^

	MCG	SCG
TPC mg GAE/100 mg	22.8 ± 4.3	72.5 ± 1.7^b^

Values presented are mean ± SD done in triplicates

^a^Concentration of the extracts used were 0.1 g/ml

^b^p < 0.001 vs MCG

*MCG* multi clove garlic, *SCG* single clove garlic, *GAE* gallic acid equivalent

### Serum biochemical markers

CCl_4_-induced hepatotoxicity in rabbits was demonstrated in the results obtained from liver function tests. An increase in serum levels of the enzymes AST (*p* < 0.01), ALT (*p* < 0.0001), ALP (*p* < 0.001) and TB (*p* < 0.001) were reported; while TSP was found to decrease insignificantly (Table 3). The orally administrated MCG significantly decreased ALT, ALP, and TB (*p* < 0.01), while activity of AST was insignificantly decreased in comparison to CCl_4_-treated rabbits. Rabbits from the TS group exhibited a much better recovery in body weight than the TM group. In addition, the TS group exhibited significant reduction of AST (*p* < 0.05), ALP (*p* < 0.001), ALT and TB (*p* < 0.01) activities. Level of TSP in both groups treated with garlic extracts caused an insignificant increase in comparison to the toxic test (T) group (Table 3).

**Table 3:** Effect of SCG and MCG extracts on the body weight and serum biochemical markers on CCl_4_ induced hepatoxicity in rabbits

	C	T	TS	TM
Body weight (g)	820.8 ± 13.45	728.3 ± 82.01	868.1 ± 43.93 ^b**^	794.7 ± 58.81
ALT (U/L)	49.5 ± 7.853	521.3 ± 64.15^a****^	296.5 ± 68.46^b**^	275.7 ± 101.3^b**^
AST (U/L)	45.5 ± 8.347	1087 ± 500.3^a**^	409.3 ± 190.9 ^b*^	635.5 ± 261.4
ALP (U/L)	99 ± 37.71	294.5 ± 17.02^a***^	137 ± 34.88^b***^	170.3 ± 61.22^b**^
TB (μmol*/L)*	5.625 ± 0.750	65.0 ± 25.47^a***^	16.33 ± 6.944^b**^	22.25 ± 9.743^b**^
TSP (g /L)	62 ± 5.888	52.5 ± 2.082	66.75 ± 10.87	60.5 ± 6.807

Each value presented is mean ± SD (*n* = 6) of three repeated set of the experiments

*C* control, *T* CCl_4_, *TS* CCl_4_ + SCG, *TM* CCl_4_ + MCG

*AST* aspartate amino transferase, *ALT* alanine aminotransferase, *ALP* alkaline phosphatase, *TB* total bilirubin, *TSP* total serum protein

*p < 0.05; **p < 0.01; ***p < 0.001; ****p < 0.0001

^a^vs C group, ^b^vs T group

### Antioxidants markers

The hepatotoxicity seen in the homogenized liver of rabbits treated with CCl_4_ was due to the disrupted levels of non-enzymatic and enzymatic antioxidants. The levels of MDA in CCl_4_ treated rabbits increased significantly, (*p* < 0.01) when compared with the control group. However, GSH level was significantly reduced (*p* < 0.05) (Table 4).

**Table 4:** Levels of lipid peroxidation, glutathione and total protein in liver homogenate of rabbits treated with SCG and MCG post CCl_4_ -induced hepatotoxicity

	C	T	TS	TM
MDA (nmole/mg tissue)	3.275 ± 0.97	8.53 ± 2.56 ^a**^	2.133 ± 1.22 ^b***^	2.63 ± 0.91 ^b**^
GSH (nmole/mg tissue)	4.628 ± 1.06	2.11 ± 1.44 ^a*^	2.53 ± 0.71	3.16 ± 1.15
TP (mg/g)	55.38 ± 4.39	72.25 ± 3.57 ^a*^	27.05 ± 7.1 ^b****^	30.7 ± 11.24 ^b****^

Each value presented is means ± SD, (*n* = 6 at least) of three repeated set of the experiments. C: control; T: CCl_4_; TS: CCl_4_ + SCG; TM: CCl_4_ + MCG

*MDA* malondialdehyde, *GSH* glutathione, *TP* total protein

^*^p < 0.05; ^**^p < 0.01; ^***^p < 0.001; ^****^p < 0.0001

^a^vs C group; ^b^vs T group

**Fig. 1:**
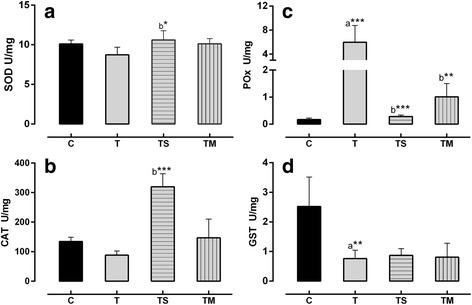
Levels of antioxidant enzymes in hepatotoxic liver of rabbits induced by CCl_4_ and treated by single clove garlic (SCG) extracts and multi clove garlic (MCG). **a**: SOD; **b**: CAT; **c**: POX; **d**: GST. Each value is mean ± SD (*n* = 6). Activity of every enzyme was expressed as U/ mg protein. C: Control, T: CCl_4_; TM: CCl_4_ + MCG, TS: CCl_4_ + SCG*.* ^*^: *P* < 0.05; ^**^: *P* < 0.01; ^***^: *P* < 0.001. ^a^: vs C; ^b^: vs T group

A significant elevation in activities of SOD (*p* < 0.05) and CAT (*p* < 0.001) was noted in the TS group (Fig. 1a and b). The level of POx in Toxic (T) group was highly elevated (*p* < 0.001) when compared to the Control (C) group. However, GST levels were significantly reduced (*p* < 0.01) (Fig. 1c and d).

The protective effect of garlic showed marked improvement in liver tissue against CCl_4_ toxicity. In both groups, TM and TS, the levels of MDA (*p* < 0.001) and TP (*p* < 0.0001) were reduced significantly compared to the Toxic (T) group (Table 4). Also, activity of POx was significantly reduced in both groups (*p* < 0.01, *P* < 0.001) respectively, with a greater decrease being reported by the TS group.

### Histopathology

**Fig. 2:**
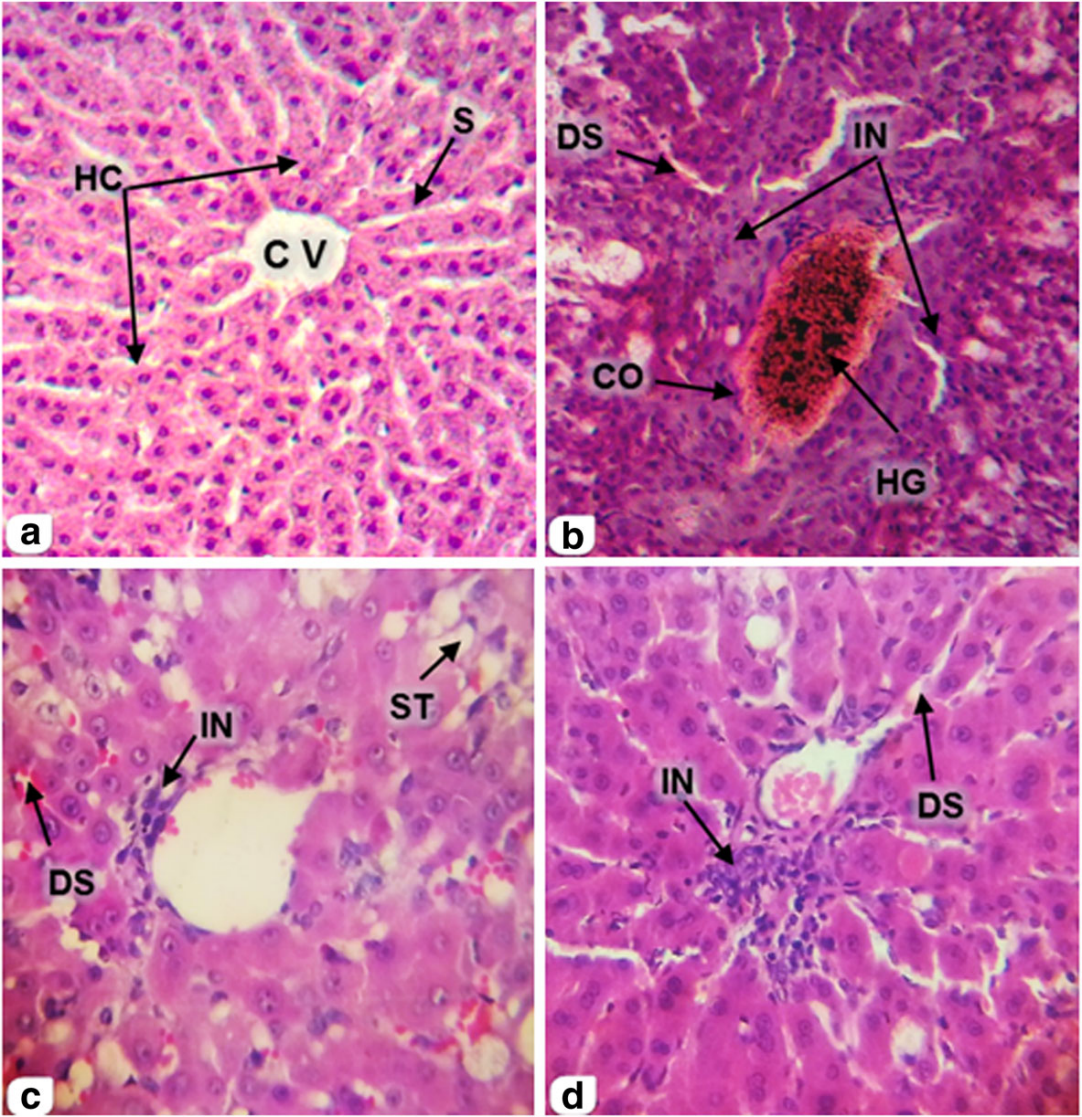
Cross sections showed the effect of SCG extracts on liver histology (pericentral vein zone) in rabbits induced hepatotoxicity by CCl_4_ (H & E, 100X). **a**: Is Control group (C) (normal control); **b**: is CCl_4_ group (T); **c**: is CCl_4_ + SCG group (TS) and **d**: is CCl_4_ + MCG group (TM). Hepatocytes (HC), Central vein (CV), Sinusoid (S), Congested central vein (CO), Deposition of haemosedrine granules (HG), dilated sinusoid (DS), Cellular inflammation (IN) and Steatosis (ST)

**Fig. 3:**
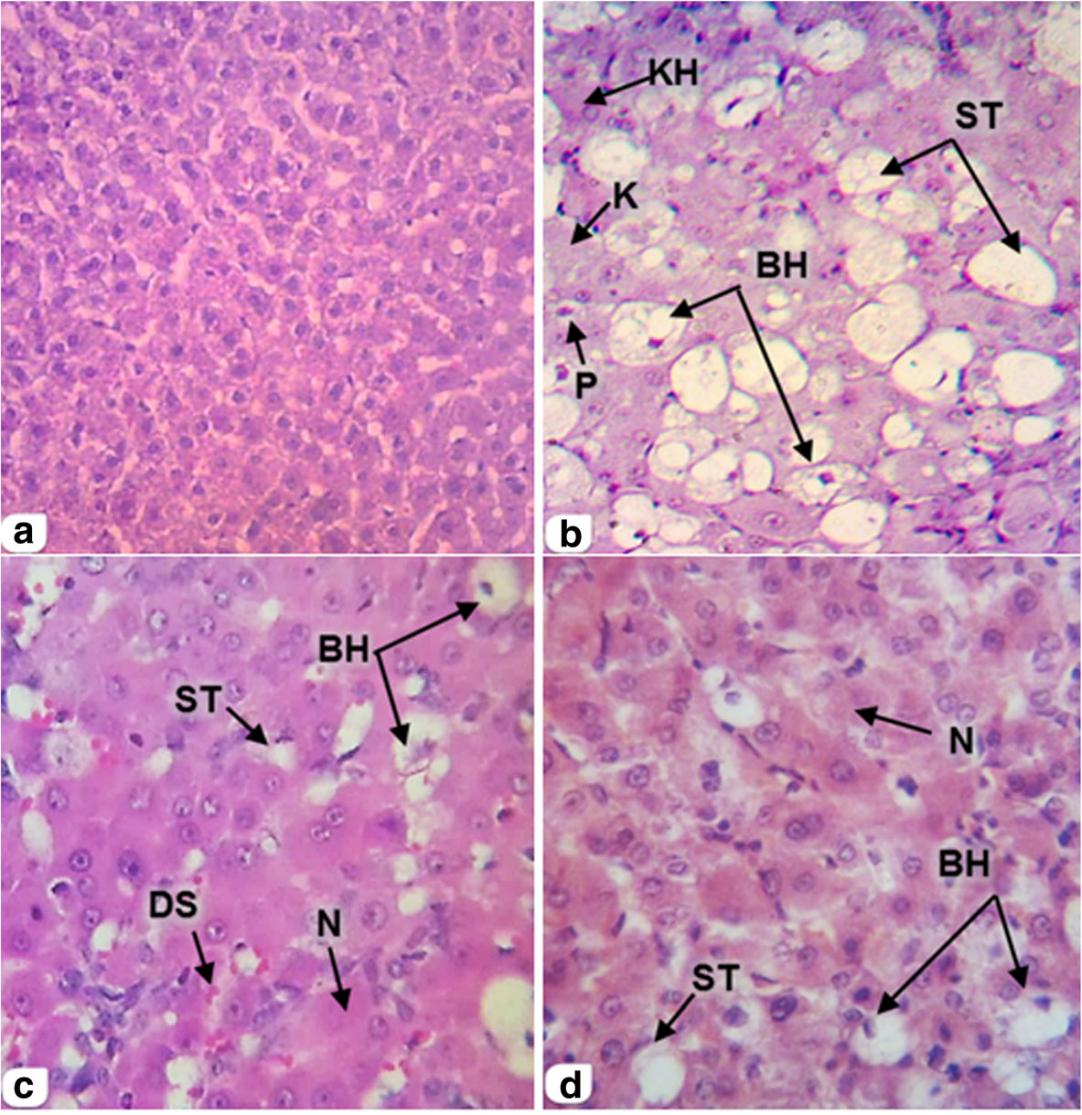
Cross sections showed the effect of SCG extracts on liver histology (Midzonal) in rabbits induced hepatotoxicity by CCl_4_ (H & E, 100X). **a**: Is Control group (C) (normal control); **b**: is CCl_4_ group (T); **c**: is CCl_4_ + SCG group (TS) and **d**: is CCl_4_ + MCG group (TM). Karyolised (K), Pyknosis (P), Karyorrhexis (KH), Steatosis (ST), Ballooned hepatocytes (BH), Dilated sinusoid (DS) and Necrosis (N). (DS) and Necrosis (N)

Histological analysis provided support to the data obtained by biochemical analysis and liver antioxidant status. In the liver of normal control animals (C) there are no pathological abnormalities with the liver sections exhibiting normal central vein and hepatic cells, i.e., a well-preserved cytoplasm, a prominent nucleus and nucleolus (Figs. 2a, 3a). Rabbits administrated orally with 0.3 ml/kg CCl_4_ i.e., the (T) group, revealed moderate to severe histopathological changes in the liver tissue. Histopathological analysis of the liver sections of CCl_4_-treated animals showed several centrilobular necrosis, hepatocyte ballooning, and infiltration of inflammatory cells into the portal tract and sinusoid (Figs. 2b and 3b).

Post treatment, rabbits of the TS and TM groups that received 0.8 g/kg SCG and MCG respectively, exhibited reversal in the appearance of hepatic lesions produced by CCl_4_ (Figs. 2c–d and 3c, d). This was evident from the absence of cellular necrosis and inflammatory infiltrates in liver sections of rabbits treated with SCG or MCG.

All scores of quantitative estimations in the T group were significantly higher than the normal control group (C); the lesions were observed as dilated sinus and cellular inflammation (*p* < 0.01), hepatocyte necrosis, steatosis and congestion (*p* < 0.001), ballooned hepatocytes (*p* < 0.0001) indicating that CCl_4_-induced severe damage to the hepatic cells. In contrast, rabbits treated with SCG showed significant reduction scores of dilated sinus and hepatocyte necrosis (*p* < 0.05), ballooned hepatocytes and steatosis (*p* < 0.01) as compared with T group. (Table 5 and Figs. 2c and 3c). Also, rabbits treated with MCG showed significant reduction in scores of dilated sinuses, ballooned hepatocytes, hepatocyte necrosis and steatosis (*p* < 0.05) as compared with T group (Table 5 and Figs. 2d and 3d).

**Table 5:** In situ evaluation of the effect of SCG and MCG on liver treated with SCG and MCG post CCl_4_ -induced hepatotoxicity

	C	T	TS	TM
Cellular inflammation	ــ	+++	++	++
	0.0	4.25 ± 2.63^a**^	2.25 ± 0.96	2.50 ± 1.29
Necrosis	ــ	++++	++	++
	0.0	5.25 ± 2.06^***^	2.25 ± 0.50^b*^	2.75 ± 0.96^b*^
Congestion	+	++++	++	++
	1.25 ± 0.50	36.12 ± 15.45^a***^	20.50 ± 5.75	18.25 ± 2.5
Ballooned hepatocytes	+	++++	+++	+++
	3.25 ± 1.89	39.50 ± 5.45^a****^	22.50 ± 8.96^b**^	26.0 ± 5.1 ^b*^
Vasodilation	+	++++	++	++
	1.75 ± 0.50	6.0 ± 2.16^a**^	2.50 ± 1.29^b*^	2.75 ± 1.71^b*^
Steatosis	ــ	++++	++	++
	0.0	22 ± 8.49^a***^	6.5 ± 3.11^b**^	8.5 ± 4.51^b*^

Results presented are means ± SD; (*n* = 6)

*C* Normal control, *T* CCl_4_, *TS* CCl_4_ + SCG, *TM* CCl_4_ + MCG

Damages graded as follows: -, absent; +, trace (1–25%); ++, mild (25–50%); +++, moderate (50–75%); ++++, severe (75–100%)

^*^ *p* < 0.05; ^**^ *p* < 0.01; ^***^ *p* < 0.001; ^****^ *p* < 0.0001

^a^vs C group; ^b^vs T group

## Discussion

Although most of the antioxidant activities from plant sources are derived from phenolic compounds, these effects do not always correlate with the presence of large quantities of phenols. The antioxidant activity of phenolic compounds is mainly due to their redox properties, allowing them to act as reducing agents, hydrogen donors, and singlet oxygen quenchers [39]. Investigation of garlic extracts for total phenolic content (TPC) showed that SCG has greater than 3 folds the amount of TPC when compared to MCG. These results are in agreement with Othman et al., [40]. The decrease in TPC is most probably caused by the increase of sulfur compounds and terpenoid substances present in the essential oil of MCG bulbs [41]. The presence of a single bulb in SCG could result in accumulation of higher contents of TPC resulting in an increase in its redox activity.

The evaluation of the antioxidant activities of different plant products was measured using numerous in vitro assays. However, each of these tests is based on their ability to scavenge free radicals or by the inhibition of lipid peroxidation [42]. DPPH assay is one of the most widely used methods for screening antioxidant activity of plant extracts. DPPH is stable and produces violet color in methanol solution. With the addition of the extract, a hydrogen atom is donated, giving rise to the reduced form of DPPH, thereby producing violet color [43]. DPPH scavenging activity of both SCG and MCG extracts exhibited lower activity than ascorbic acid. However, SCG showed ~15% higher activity than MCG. The scavenging ability against superoxide radical (O_2_^•-^) and H_2_O_2_ were almost similar for both extracts. O_2_^•-^ is one of the precursors of HO^•^ or singlet oxygen and can produce other kinds of oxidizing agents, where it indirectly initiates lipid peroxidation and magnifies cellular damage. H_2_O_2_ a non-free radical species, is the source of the very toxic HO^•^ radical which can cross membranes and may slowly oxidize a number of cell compounds [44]. Our results on scavenging activity indicate that garlic has a remarkable potency to donate electrons to reactive free radicals, converting them into more stable non-reactive species and terminating the free radical chain reaction.

Oxidative toxicity induced by CCl_4_ in the liver of rabbits belonging to the T group was confirmed by the parameters such as body weight, liver function tests, oxidative stress and some histological responses. The rise in ALT activity is almost always due to hepatocellular damage and is usually accompanied by a rise in AST and ALP [30]. The serum levels of the liver enzymes, AST, ALT and ALP was found to be higher in group T when compared to group C (Table 3), indicating greater oxidative hepatotoxicity in the former group. These results are similar to several studies done on CCl_4_ induced hepatotoxicity [45]. The mechanism of CCl_4_ injury involves oxidative damage by metabolism of CCl_4_ to CCl_3_^•-^ in hepatocytes, resulting in cellular membrane degeneration, increased permeability, cell death and leakage of ALT, AST and ALP into the serum [46]. It is indicative of cellular damage and loss of functional integrity in liver [47]. Although concomitant treatment with garlic extracts resulted in a significant decrease in the level of serum enzymes (Table 3), the TS group showed reduction in AST and ALP compared with TM group. This suggests that both SCG and MCG have ability to abolish alterations induced by CCl_4_ in the activities of ALP, ALT and AST; with SCG having greater ability than MCG; thus, possessing preventive action.

Liver injury is associated with decreased rate of hepatic synthesis of the essential proteins in both humans and animals [48]. Accordingly, total serum protein (TSP) was found to decline in T group. Hence, it can be deemed as a useful index of cellular dysfunction in liver toxicity as clearly shown in our results (Table 3). In contrast, the administration of SCG extract showed recovery of TSP level better than TM and control group. This suggests that SCG promotes protein synthesis and plays an axial role in hepatoprotective activity, which in turn enhance proliferative process and the production of hepatocytes [49].

Increased bilirubin in serum or tissue is indicative of obstruction in the excretion of bile as a result of liver damage. T group exhibited a massive serum bilirubin elevation. However, the significant decline in bilirubin levels in animals of TS group indicates a higher protective effect of SCG, which could recover 75% of the bilirubin even more than the TM recovery of 65% (Table 3). This recovery to normal may contribute in the healing of hepatic parenchyma and the regeneration of hepatocytes [50].

Lipid peroxidation is the main sign of oxidative toxicity caused due to induction of oxidative degradation of membrane lipids rich in polyunsaturated fatty acids forming malondialdehyde (MDA) [15]. The T group treated with CCl_4_ alone, showed maximum elevation in MDA levels, approximately 2-fold compared to the C group. This is indicative of oxidative hepatotoxicity causing an alteration in structure and function of cellular membranes [51], thereby resulting in excessive production of free radicals [52]. Both TS and TM groups exhibited significantly lower levels of MDA, presumably due to impaired initiation and propagation of the peroxidative process that could be linked possibly to higher TPC and antioxidant activity in the garlic extracts (Table 4). MDA levels do not appear to be significantly different when compared to TS and TM. Hence, the protective mechanism of garlic against CCl_4_-induced hepatotoxicity is either by decreasing the metabolic activation of CCl_4_ or by acting as a chain breaking antioxidant for scavenging free radicals, or by a combination of both effects [53].

GSH, a key antioxidant, is an important constituent of intracellular protective mechanisms against oxidative stress due to its sulfhydryl group that binds to a variety of electrophilic radicals and metabolites. Depletion of GSH level in both TS and TM indicate its role in detoxification. Indeed, glutathione depletion increases the sensitivity of cells to various aggressions and has several metabolic effects [54]. The main detoxification enzyme of the liver involved in reactions of phase I is CY-P-450 that consumes GSH. This explains the greater reduction in GSH reported in TS when compared to that of the TM group.

The increase in POx activity reported in T group is indicative of its involvement in the detoxification phase of liver cells. A significant decrease in its activity was seen in both TS and TM groups, with the activity in the TS declining almost to the levels reported for C group.

Free radical scavenging enzymes such as SOD and CAT are the major defense enzymes against oxidative injury [55]. SOD converts the highly reactive superoxide radical to H_2_O_2_, which in turn is metabolized either by CAT or GPx to water and O_2_ thus protecting the cell from oxidative damage that would have resulted from H_2_O_2_ and hydroxyl radical [33]. SOD and CAT levels were significantly elevated in both TS and TM groups. In TS, elevation in CAT and reduction in POx activity was demonstrated implying that hepatic cells in SCG treated group was able to detoxify H_2_O_2_ by the CAT scavenging system rather than POx. This indicates that SCG has the ability to restore and/or maintain the activities of hepatic CAT during oxidative toxicity. In contrast, the accumulation of MDA in TM group corresponded well with the reduction in CAT activity via inhibition of protein synthesis [56].

Glutathione-s-transferases (GST) are a family of enzymes that catalyze the conjugation of the tripeptide glutathione to endogenous and xenobiotic substrates causing metabolism followed by detoxification of these compounds [57]. Activities of cellular GSTs were decreased significantly in liver tissue of T group due to depletion of the enzyme as a result of permeability disruption by oxidative radicals [58], or inhibitory effect on the active site of the enzyme by free radicals (Table 4).

The histological evaluation of liver sections supports the results obtained from serum enzyme assays. The changes noted in hepatic cells by free radicals resulting from CCl_4_ toxicity is due to cellular injury occurring by alteration in membrane permeability. The marked changes seen were: dilation and congestion of the hepatic vascularity, ballooned hepatocytes, inflammation, necrosis and steatosis were statistically evaluated. The reactions elicited is to eliminate or limit the spread of injurious agents as well as to remove the consequent cells and tissue. Oxygen derived metabolites are released from activated neutrophils and macrophage and include superoxide, hydrogen peroxide, and hydroxide radicals. These leads to endothelial cell damage, increased vascular permeability, progressive degenerative action of intracellular enzymes, metabolic disturbances and inhibition of protein synthesis for the growth and maturation of the liver and finally hepatocyte necrosis. The latter is either a direct cause or a means of reversible injury.

Vascular disorders of the liver which were noticed in the present study may be due to heart failure, the most common cause of liver congestion; and sinusoid dilation causing increase venous blood in organs because of obstruction to the venous outflow. This increases blood pressure in the veins and capillaries. The liver is an important organ for storage and degradation of iron leading to appearance of hemosiderin granules in hepatic vessels. Accumulation of fat in liver results in steatosis, a condition clearly presented in the T group and markedly reduced in the TS and TM groups, the former exhibiting maximum reduction. Impairment in free fatty acids metabolism leads to net accumulation of triglyceride within the liver. Ballooned hepatocytes thus seen were formed due to excess water accumulation inside cytoplasmic vacuoles as a result of active cell membrane transport failure [59]. This explains the increase in numbers of ballooned hepatocytes in the toxic group. Recovery was noted with the administration of SCG and MCG. Microscopic examinations showed marked improvement in some histopathological changes of liver in T group by concomitant treatment with garlic in TS and TM groups. However, recovery in all scores of histopathological changes by SCG treatment was better than MCG.

Our observations were supported by other studies suggesting that garlic exerts protective effects against many chemical agent induced hepatotoxicity. This may be attributed to the presence of numerous antioxidant compounds that scavenge superoxide anion and hydroxyl radicals and also by enhancing the activity of liver SOD and reducing liver MDA contents. This supports the idea that *A. sativum* is a good natural antioxidant source and protects cellular membranes and lipoproteins against peroxidation.

## Conclusion

Our data exhibited that SCG possessed greater antioxidant potential than MCG. The ethanolic extract of SCG was linked to a greater decrease in oxidative toxicity. It could be due to the higher TPC in SCG than MCG. The removal of H_2_O_2_ was dependent on CAT activity rather than POx in the TS group. SCG can regulate lowering of free radicals, improve liver and cholestatic biomarkers, ameliorate hepatic marker enzymes, reduce severity of fibrosis and normalize the hepatocyte architecture. The hepatoprotective effect of SCG demonstrated in this study may enhance its therapeutic benefits as a potential preventive intervention for free radical-mediated liver injury.

## Additional files

Additional file 1: Figure S1. Pictures of peeler and unpeeled Single clove garlic (SCG) used in this study. (TIFF 2454 kb)

Additional file 2: Figure S2. Pictures of peeler and unpeeled Multi clove garlic (MCG) used in this study. (TIFF 2379 kb)

## Abbreviations

ALP: Alkaline phosphatase
ALT: Alanine aminotransferase
AsA: Ascorbic acid
AST: Aspartate aminotransferase
CAT: Catalase
CCl_4_: Carbon tetrachloride
DPPH: 2,2-diphenyl-1-picrylhydrazyl
GSH: Glutathione
GST: Glutathione-s-transferase
MCG: Multi clove garlic
MDA: Malondialdehyde
POX: Peroxidase
ROS: Reactive oxygen species
SCG: Single clove garlic
SOD: Superoxide dismutase
TB: Total bilirubin
TM: Group treated by MCG
TP: Total tissue protein
TS: Group treated with SCG
TSP: Total serum protein.
