# Supplementary figures and images for: Hepatoprotective and antioxidant effects of single clove garlic against CCl4-induced hepatic damage in rabbits
Source: BMC Complement Altern Med. 2017 Aug 17;17:411. doi: 10.1186/s12906-017-1916-8 (PMC5561638; doi:10.1186/s12906-017-1916-8)

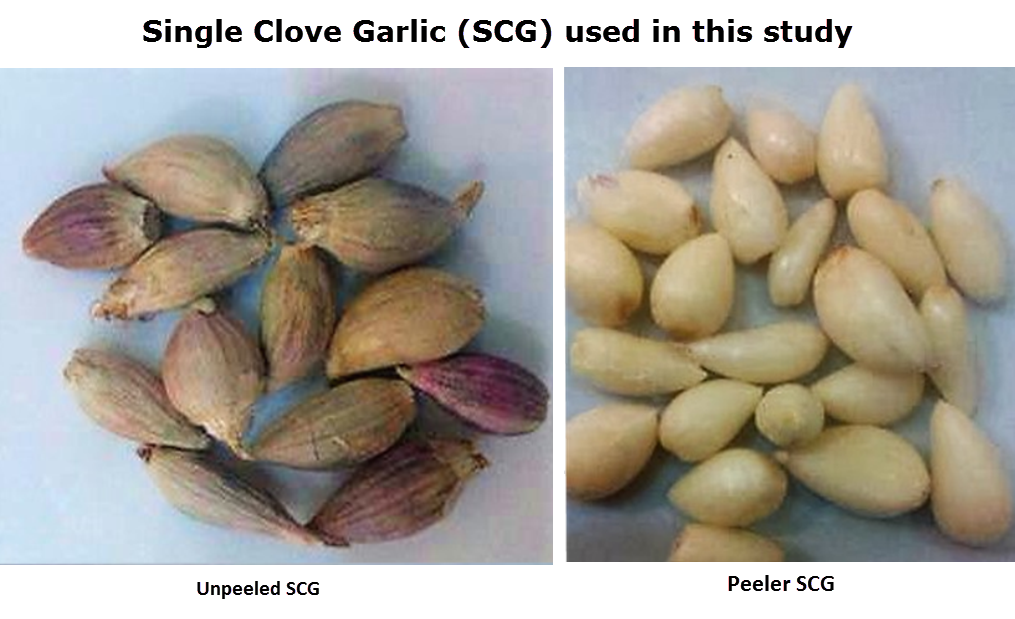

Supplement: Supplementary file 1 — Pictures of peeler and unpeeled Single clove garlic (SCG) used in this study. (TIFF 2454 kb) [file 12906_2017_1916_MOESM1_ESM.tif]

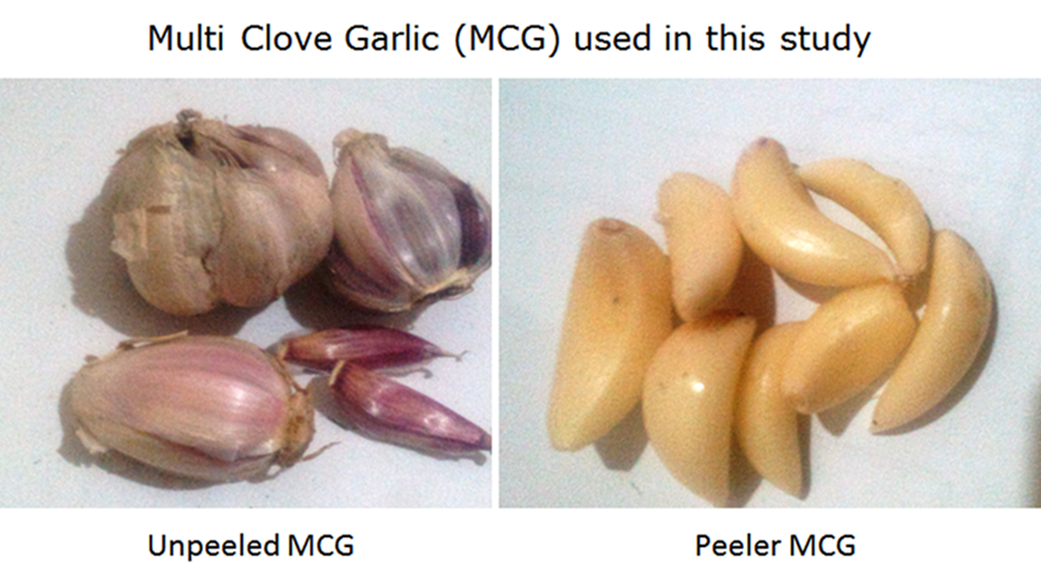

Supplement: Supplementary file 2 — Pictures of peeler and unpeeled Multi clove garlic (MCG) used in this study. (TIFF 2379 kb) [file 12906_2017_1916_MOESM2_ESM.tif]
